# The Association Between Atopic Dermatitis and Inflammatory Bowel Disease Risk: A Meta‐Analysis of Longitudinal Studies

**DOI:** 10.1002/jgh3.70077

**Published:** 2024-12-12

**Authors:** Pengfei Yu, Li Lin, Kaiyuan Xue, Jingwen Yang, Shanshan Wang, Yuepeng An, Suqing Yang

**Affiliations:** ^1^ The First Clinical College of Heilongjiang University of Traditional Chinese Medicine Harbin People's Republic of China; ^2^ Department of Dermatology, The First Affiliated Hospital of Heilongjiang University of Traditional Chinese Medicine Harbin People's Republic of China

**Keywords:** atopy, bowel disease, Crohn's disease, eczema, ulcerative colitis

## Abstract

**Aims:**

This review aimed to investigate whether atopic dermatitis (AD) increases the risk of inflammatory bowel disease (IBD) by analyzing data from longitudinal studies.

**Methods:**

Cohort and case–control studies evaluating the association between AD and the risk of IBD, Crohn's disease (CD), or ulcerative colitis (UC) were included. Literature searches were conducted in PubMed, CENTRAL, Embase, Scopus, and Web of Science databases up to April 15, 2024.

**Results:**

A total of eight retrospective cohort studies comprising 61 190 816 participants were included. Meta‐analysis revealed that AD significantly increased the risk of IBD (OR: 1.37, 95% CI: 1.31–1.43) without statistical heterogeneity. Further pooled analysis showed that AD was a significant risk factor for CD (OR: 1.51, 95% CI: 1.31–1.76) and UC (OR: 1.33, 95% CI: 1.13–1.56), with high inter‐study heterogeneity (*I* ^2^ = 83% and 89%, respectively). Sensitivity analyses confirmed the robustness of the results.

**Conclusion:**

AD is associated with an increased risk of IBD, significantly elevating the risk of both CD and UC.

## 1 Introduction

Atopic dermatitis (AD) or eczema is a chronic inflammatory skin disease that causes dry, itchy, and inflamed skin. Onset occurs most commonly in childhood and about 60% of patients exhibit remission in adolescence [1]. However, a large proportion of patients continue to have symptoms during teenage and adulthood, and for some, the symptoms may first appear later in life [2]. Recent data suggests that significant regional variation exists in the prevalence of AD. One‐year prevalence is about 1.2% in Asia–17.1% in Europe for adults while 0.96%–22.6% in children in Asia are affected by the disease [3].

Inflammatory bowel disease (IBD) is another major healthcare problem consisting of two prominent phenotypes, namely Crohn's disease (CD) and ulcerative colitis (UC). About 0.3% of the world population suffers from IBD contributing to significant gastrointestinal (GI) morbidity [4]. The disease is characterized by chronic inflammation of the GI tract due to an unknown etiology with possible links to geographical location, inappropriate diet, genetics, and an inappropriate immune response [5]. Investigations have noted that IBD symptoms are not restricted to a dysregulated immune response in the GI tract but also have several extra‐intestinal manifestations like inflammatory skin lesions, arthritis, spondylarthropathy, episcleritis, uveitis, and primary sclerosing cholangitis [6]. Similarly, a large number of systemic diseases like alopecia, vitiligo, IBD, and attention‐deficit/hyperactivity disorder have also been linked with AD [7].

Over the past decade, research has shown that AD and IBD share several genetic, environmental, and microbial risk factors suggesting a bidirectional relationship between the two disease entities [8, 9, 10]. While several studies [11, 12, 13] have examined the relationship between the two, evidence remains inconsistent. Further, previous systematic reviews [10, 14, 15] have been predominantly based on cross‐sectional and unadjusted data producing low‐quality evidence. Hence, the purpose of this updated systematic review was to examine the longitudinal risk of IBD in patients with AD by pooling high‐quality evidence only from cohort and case–control studies.

## 2 Methods

The study followed the PRISMA [16] reporting guidelines and was registered on PROSPERO. The registration number provided was CRD42024534833. The review did not require ethical approval as data were analyzed from already published studies.

### 2.1 Search Strategy

We searched Medline using PubMed, CENTRAL, Embase, Scopus, and Web of Science electronic repositories. Two authors searched for all English language publications available before 15th April 2024. There was no limit on the initial publication date. Results were limited to human studies only. The medical librarian helped the study authors in conducting the search.

The reviewers used specific keywords to identify relevant studies. The following search string was formulated and replicated across databases: (((atopic dermatitis) OR (eczema)) OR (atopy)) AND (((ulcerative colitis) OR (Crohn's disease)) OR (inflammatory bowel disease)). Reference lists from reviews and included studies were also additionally searched. We also searched Google Scholar to identify studies from the gray literature. Results from all databases were combined after completion of the search to generate a master database. Filtration was done first by deduplication, then by primary and secondary screening. Primary screening involved the elimination of nonrelevant studies by reading the titles and abstracts. Important studies were identified, and their full texts were retrieved. These underwent secondary screening by examination of full texts. Disagreements were discussed by including a third author and resolved. If any outcome data were missing or not reported properly, the corresponding author of the article was contacted once via e‐mail.

### 2.2 Inclusion Criteria

Cohort and case–control studies that were conducted on the general population (adult or pediatric) and examined the longitudinal association between AD and subsequent risk of IBD were included in this review. We included studies that reported multivariate‐adjusted associations only.

Studies not reporting on IBD, UC, or CD, not reporting separate data for AD, and cross‐sectional studies were excluded. For articles in which a sample was repeated, the article with the largest sample size was included.

### 2.3 Data Extraction and Study Quality

A pre‐defined form was used to collect information on study characteristics (author, year of publication, database, location, study design, sample size), assessment of AD and IBD, demographic details of the sample, the proportion of AD and IBD in the sample, factors adjusted in the multivariate analysis, follow‐up and effect size.

The authors chose to use the Newcastle‐Ottawa Scale (NOS) to assess study quality [17]. Every study was assessed for the selection of cohorts, comparability of the study and control groups, outcome measurement, and availability of follow‐up and awarded points ranging from zero to nine. Two authors conducted the bias assessment and any differences of opinion were resolved by involving a third author.

### 2.4 Statistical Analysis

Owing to the limited incidence of IBD in the cohorts, hazard ratios, risk ratios (RR), and incident ratios were considered as odds ratios (OR) [18]. The impact of AD on the risk of IBD was expressed as OR with 95% confidence intervals (CI). All analysis was done in “Review Manager” (RevMan, version 5.3; Denmark; 2014). Data were combined utilizing the generic inverse variance function of Review Manager. Separate analysis was conducted for unclassified IBD, CD, and UC. Significant heterogeneity was defined by the *I* ^2^ statistics ≥ 50% and/or *p* < 0.10 of the Cochrane Q test. We chose a random effect model for all meta‐analyses due to the expected methodological heterogeneity in studies, regardless of the results of statistical heterogeneity. Funnel plots were used to examine publication bias for meta‐analysis with > 5 studies. To investigate the impact of individual studies on the overall risk summary, we performed a sensitivity analysis by removing one study at a time. Subgroup analysis was performed for the analysis of UC and CD for the following variables: population (adult vs. pediatric), method of assessment of AD, proportion of AD in the population, adjustment for asthma or other allergies, and NOS score.

## 3 Results

### 3.1 Search Outcomes

**FIGURE 1:**
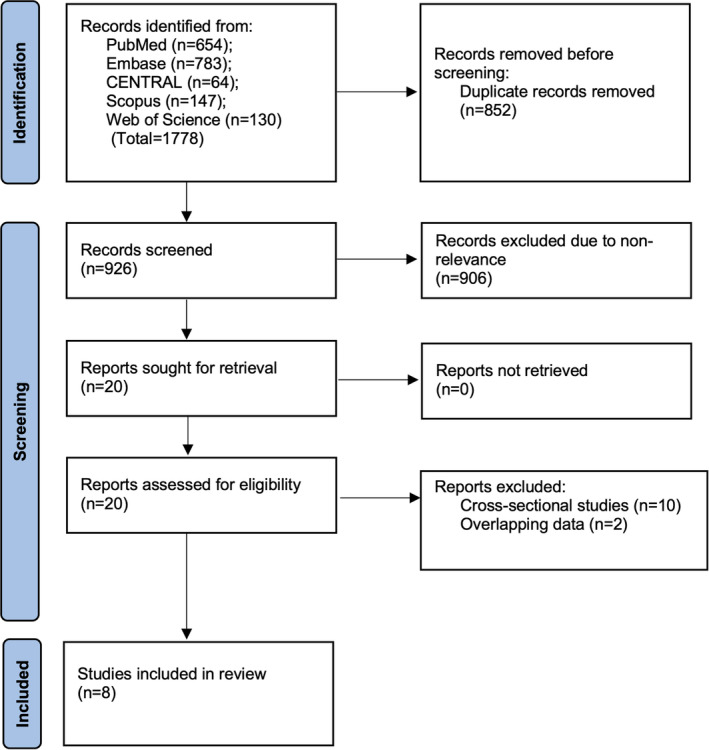
PRISMA flowchart—Study selection and literature search results.

On completion of the search, the master database had 1778 articles. Among these 852 studies were duplicates and excluded. 926 articles underwent primary screening and the reviewers selected 20 for secondary screening. The inter‐reviewer agreement was high (kappa = 0.85). After excluding cross‐sectional studies and articles with overlapping data, the authors found eight studies [11, 12, 13, 19, 20, 21, 22, 23] suitable for this review (Figure 1). An additional search of reference lists and gray literature did not reveal additional studies. For the final included studies, there were no disagreements among reviewers.

### 3.2 Details of Studies

Information extracted from studies can be found in Table 1. All were retrospective cohort studies examining the longitudinal risk of IBD following exposure to AD. The retrieved studies mostly used insurance or national health registers to analyze the data. Overall, the eight studies examined a combined sample size of 61 190 816 participants. One study reported two cohorts (one adult and the other pediatric). Assessment of AD and IBD was primarily by medical codes across studies. Only one study reported a diagnosis of AD by a dermatologist while another used questionnaires to elicit the diagnosis of AD. The percentage of AD varied between 0.19 to 20.9%. The incidence of IBD ranged from 0.05% to 0.43%. The majority of studies adjusted for age and gender in the multivariate analysis. Other covariates differed among studies. Follow‐up was also variable and ranged from 4 to up to 19 years in the included studies. Two studies were awarded seven points on NOS. All remaining studies achieved eight points on NOS.

**TABLE 1:** Characteristics of included studies.

Study	Database	Design	Assessment of AD	Assessment of IBD	Sample size	Male (%)	Age (years)	% with AD	% with IBD	Adjusted factors	Follow‐up	NOS score
Lerchova 2024 [13]	All Babies in Southeast Sweden and Norwegian mothers, father, and child cohorts	R	Questionnaires	ICD codes	83 311	51.2	3	20.9	0.4	Sex, parental history of atopy, parental country of birth, parental IBD, maternal education level, and smoking during pregnancy	13.5–19.2 years	7
Fuxench 2023 [19]	Optum Insurance Administrative database, USA	R	ICD codes	ICD codes	40 141 017	49.2	46.7	1.5	NR	Residence location, age, sex, asthma, seasonal allergies, and hypertension	NR	7
Fuxench 2023 [20]	The Health Improvement Network, UK	R	ICD codes	ICD codes	3 303 971	44.8	48	18.9	NR	Age, sex, Townsend index, history of asthma or allergic rhinitis, use of systemic steroids, body mass index, smoking, and alcohol use	5 [2–10] years	8
	The Health Improvement Network, UK	R	ICD codes	ICD codes	2 218 460	51.8	4–9	18.5	NR	Age, sex, Townsend index, history of asthma or allergic rhinitis, use of systemic steroids, and body mass index	5 [2–9] years	8
Lusignan 2022 [22]	Oxford–Royal College of General Practitioners Research and Surveillance Centre primary care database, UK	R	Read codes	Read codes	868 545	20	27	1.72	0.17	Age, sex, deprivation quintile, ethnicity, body mass index category, smoking status, alcohol use category, number of visits in the 2 years before diagnosis, type 2 diabetes, hypertension, atrial fibrillation, angina, acute myocardial infarction, stroke, heart failure, chronic liver disease, dementia, chronic obstructive pulmonary disease, chronic kidney disease stage 3–5, malignancy, depression, and hyperlipidemia	4.1 years	8
Weng 2020 [21]	National Health Insurance Research Database, Taiwan	R	ICD codes	ICD codes	401 170	23	47.5	9	0.05	Age, sex, and comorbidities, including allergic rhinitis, asthma, infections, hypertension, diabetes, cardiovascular disease, autoimmune diseases, and psoriasis	6.6–8.1 years	8
Soh 2020 [23]	National Health Insurance, Korea	R	ICD codes	ICD codes	9 923 521	NR	NR	0.4	0.07	Age, sex, residence, smoking history, alcohol consumption, regular exercise, income, body mass index, diabetes, hypertension, and dyslipidemia	7.3 years	8
Egeberg 2017 [12]	Nationwide Danish registers	R	By dermatologist	NR	3 595 006	48.9	NR	0.19	0.3	Age, sex, socioeconomic status, smoking, alcohol abuse, and healthcare consumption	Up to 4 years	8
Schmitt 2016 [11]	German National Health Insurance	R	ICD codes	ICD codes	655 815	50.5	NR	7.6	0.43	Age, sex, socioeconomic status, access to health care, and health care utilization behavior)	Up to 5 years	8

Abbreviations: AD, atopic dermatitis; IBD, inflammatory bowel disease; ICD, international classification of diseases; NR, not reported; NOS, Newcastle‐Ottawa scale; R, retrospective.

### 3.3 Meta‐Analysis

**FIGURE 2:**
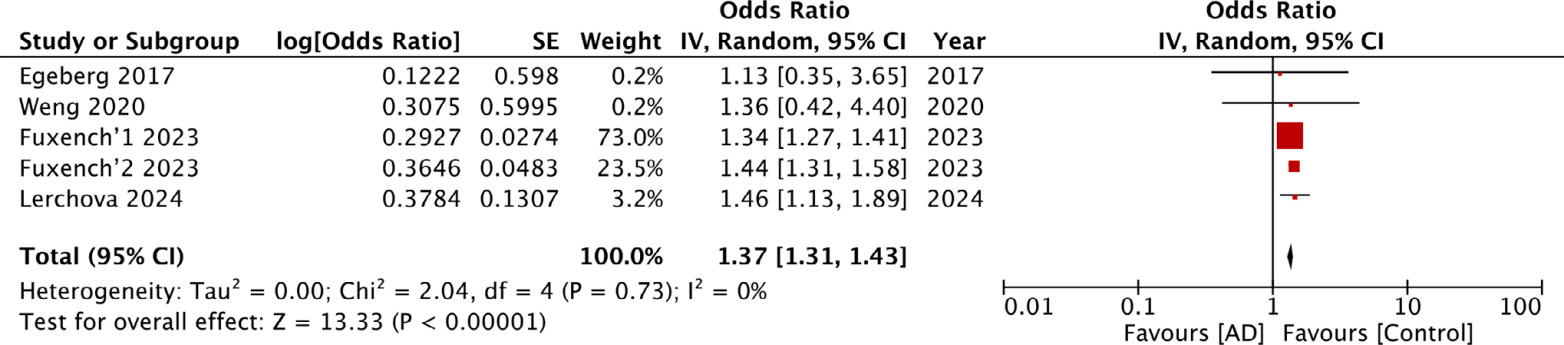
In the forest plot, atopic dermatitis (AD) is identified as a significant risk factor for inflammatory bowel disease (IBD).

**FIGURE 3:**
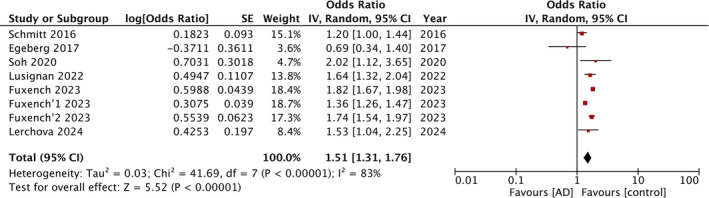
In the forest plot, atopic dermatitis (AD) is identified as a significant risk factor for Crohn's disease (CD).

**FIGURE 4:**
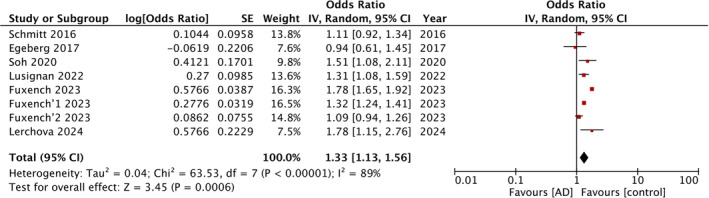
In the forest plot, atopic dermatitis (AD) is identified as a significant risk factor for ulcerative colitis (UC).

Four studies corresponding to five cohorts reported data on overall IBD risk. The meta‐analysis found that AD was a significant risk factor for IBD with OR: 1.37 and 95% CI: 1.31, 1.43 (Figure 2). No statistical heterogeneity was noted. Seven studies with eight cohorts examined the risk of CD and UC with AD. Pooled analysis showed that exposure to AD was a significant risk factor for both CD (OR: 1.51 95% CI: 1.31, 1.76) (Figure 3) and UC (OR: 1.33 95% CI: 1.13, 1.56) (Figure 4). Both meta‐analyses had high inter‐study heterogeneity with *I* ^2^ = 83% and *I* ^2^ = 89% respectively. The funnel plot (Figures S1 and S2) illustrates a symmetrical scatter distribution at the top, indicating that the included studies are robust and no publication bias has been detected.

### 3.4 Sensitivity Analysis

Results of sensitivity analysis conducted for IBD, CD, and UC are shown in Table 2. It can be seen that the significance of the results did not change for any of the meta‐analyses on the exclusion of any study. There was some variance in effect size on the exclusion of the studies. The OR for IBD ranged from 1.34 to 1.44. For CD, the OR varied from 1.46 to 1.58 while for UC it ranged from 1.24 to 1.37. The statistical heterogeneity was persistently high for the meta‐analysis of both CD and UC during the sensitivity analysis.

**TABLE 2:** Results of sensitivity analysis.

Excluded study	Odds ratio [95% confidence intervals]	*I* ^2^
Inflammatory bowel disease		
Egeberg 2017	1.37 [1.31, 1.43]	0
Weng 2020	1.37 [1.30, 1.43]	0
Fuxench'12023	1.44 [1.32, 1.57]	0
Fuxench'22023	1.34 [1.28, 1.42]	0
Lerchova 2024	1.36 [1.30, 1.43]	0
Crohn's disease		
Schmitt 2016	1.58 [1.36, 1.84]	82
Egeberg 2017	1.56 [1.35, 1.80]	84
Soh 2020	1.49 [1.28, 1.74]	85
Lusignan 2022	1.49 [1.26, 1.76]	86
Fuxench 2023	1.46 [1.25, 1.69]	73
Fuxench'12023	1.56 [1.33, 1.82]	74
Fuxench'22023	1.47 [1.23, 1.75]	84
Lerchova 2024	1.51 [1.29, 1.77]	86
Ulcerative colitis		
Schmitt 2016	1.37 [1.15, 1.62]	89
Egeberg 2017	1.37 [1.16, 1.61]	90
Soh 2020	1.31 [1.10, 1.56]	91
Lusignan 2022	1.33 [1.11, 1.59]	90
Fuxench'12023	1.33 [1.06, 1.65]	89
Fuxench'22023	1.37 [1.16, 1.63]	88
Fuxench 2023	1.24 [1.12, 1.38]	53
Lerchova 2024	1.30 [1.09, 1.53]	90

### 3.5 Subgroup Analysis

Subgroup analysis was conducted only for the meta‐analysis of CD and UC and not for IBD due to limited studies in the latter analysis. Detailed results are shown in Table 3. Results were significant across most subgroups with a few exceptions. We noted a nonsignificant association between AD and UC in the pediatric subgroup. Also, we noted no significant association between AD and CD/UC when studies did not use medical codes for identifying AD.

**TABLE 3:** Subgroup analysis.

Variable	Groups	Cohorts	CD	*I* ^2^	UC	*I* ^2^
Population	Pediatric Adult	2 6	1.72 [1.53, 1.93] 1.46 [1.21, 1.76]	0 87	1.33 [0.83, 2.14] 1.34 [1.12, 1.60]	77 90
Assessment of AD	Medical codes Other	6 2	1.56 [1.34, 1.82] 1.09 [0.50, 2.36]	86 73	1.33 [1.12, 1.59] 1.29 [0.69, 2.42]	92 76
% with AD	> 5% < 5%	4 4	1.44 [1.21, 1.71] 1.63 [1.30, 2.06]	80 62	1.23 [1.06, 1.42] 1.41 [1.10, 1.82]	69 81
Adjusted for asthma or other allergies	Yes No	3 5	1.62 [1.33, 1.98] 1.39 [1.09, 1.77]	93 61	1.38 [1.07, 1.77] 1.27 [1.07, 1.51]	96 44
NOS score	7 8	2 6	1.81 [1.66, 1.96] 1.45 [1.23, 1.70]	0 77	1.78 [1.65, 1.92] 1.22 [1.10, 1.36]	0 53

Abbreviations: AD, atopic dermatitis; CD, Crohn's disease; NOS, Newcastle Ottawa scale; UC, ulcerative colitis.

## 4 Discussion

In this study, we chose a longitudinal cohort study design because of its unique advantages in causal inference. In contrast, cross‐sectional studies, while better suited to assess prevalence and describe correlations, have limitations in causal inference. The findings of this systematic review and meta‐analysis have shown that the diagnosis of AD leads to a 37% increase in the risk of unspecified IBD later in life, and the risk can range from 31% to 43%. Classifying IBD into its two prominent phenotypes, AD was associated with a 51% increase in the risk of CD and a 33% increase in the risk of UC. The strength of this association was strong and persistent during sensitivity analysis showing that the results were not influenced by a singular study. The absence of publication bias further adds to the credibility of the results.

Our review has built upon evidence of previous systematic reviews which too have demonstrated similar results but with limited and poor‐quality data. Shi et al. [15] have earlier investigated the association between AD and IBD in their meta‐analysis but could include just four studies. They noted a significant association between AD and IBD (RR: 1.44 95% CI: 1.14; 1.82), CD (RR: 2.06 95% CI: 1.61; 2.64), and UC (RR: 1.66 95% CI: 1.23; 2.24) but on pooled analysis of unadjusted data. Another important drawback of the review was the inclusion of both cross‐sectional and cohort studies in the same meta‐analysis. Another meta‐analysis by Lee et al. [14] also mixed cross‐sectional and cohort studies and used unadjusted data to study the incidence and prevalence of IBD in AD. They found a nonsignificant risk of IBD (RR: 1.46 95% CI: 0.98; 2.17) and UC (RR: 1.11 95% CI: 0.88; 1.41) with AD but a positive association was noted between AD and subsequent risk of CD (RR: 1.28 95% CI: 1.06; 1.50) albeit with data only from three cohort studies. More recently, Lu et al. [10] pooled data from five cross‐sectional and cohort studies to show that AD was associated with an increased risk of developing both CD (RR: 1.66 95% CI: 1.50–1.84) and UC (RR: 1.95 95% CI: 1.57–2.44). Our meta‐analysis differs from these reviews. In contrast, the present study incorporated high‐quality longitudinal cohort studies, effectively addressing the limitations of unclear time series present in earlier research and enhancing the ability to infer causality. We excluded cross‐sectional studies, thereby significantly improving the reliability of our causality inferences. Furthermore, our study pooled data from 61 million participants across nine cohorts, allowing for a more comprehensive risk assessment and a thorough exploration of the causal relationship between AD and IBD with rigorous multivariate adjustments. A second important differentiating factor of this review is the use of only multivariate‐adjusted data. When examining a casual association of exposure and outcome, the importance of deliberate and rigorous control of confounders cannot be underestimated. IBD is potentially linked to various environmental risk factors, including lifestyle choices and drug exposure. In this study, we accounted for these significant confounders in our analysis, which enhances the robustness of the results and improves the accuracy of causal inferences. Inadequate control of confounding can lead to false associations which downgrade the credibility of evidence [24]. Indeed, IBD can be linked to a plethora of environmental risk factors ranging from lifestyle, hygiene, vaccinations, exposure to drugs, diet, nutritional intake, gastrointestinal pathogens, and psychosocial factors [9]. None of the included studies could optimally adjust for all risk factors in their multivariate analysis primarily due to the unavailability of data from their respective databases. However, at least partial adjustment was undertaken which improved the quality of available evidence.

A third differentiating factor of this review was the conduct of multiple subgroup analyses to explore the source of inter‐study heterogeneity which has not been conducted in any prior reviews to date. While we noted no inter‐study heterogeneity in the meta‐analysis of IBD, significant heterogeneity was seen in the analysis of CD and UC. It is important to note that although the studies included in our analysis were primarily based on retrospective cohorts, the sample sources varied across these studies, which may have influenced the results. Population characteristics, such as age, gender, and race, differed among the studies, potentially leading to variations in effect sizes. For instance, certain exposure factors might exert stronger effects on specific age groups or genders, resulting in inconsistent findings across the studies. Furthermore, discrepancies in diagnostic modalities may have significantly impacted the rates of confirmed diagnoses and the outcomes of correlation analyses for AD and IBD. While we conducted subgroup analyses to mitigate heterogeneity through stratified approaches, some heterogeneity remained. Notably, in the pediatric subgroup, we did not find a significant association between AD and UC, indicating that the impact of AD may differ by age. Similarly, the association between AD and CD/UC did not achieve statistical significance when medical codes were not employed to identify AD, further underscoring the influence of variations in diagnostic criteria on the results. Subgroup analysis based on population (adult vs. pediatric), proportion of AD in the population, adjustment for asthma or other allergies, and NOS score did not reveal major changes in the effect size except for a few variations. Limiting our data to the pediatric population, we found that while AD increased the risk of CD, no association was seen between AD and UC. However, this subgroup included only two studies, and hence the results should be interpreted with caution till the evidence is substantiated by future studies. Importantly, CD is more prevalent than UC in children [25]. Also, rectal sparing is often seen with UC in children frequently leading to misdiagnosis. UC is also indistinguishable from indeterminate colitis in children which is noted in about 33% of pediatric IBD [26]. Since data in the included studies were obtained from registries with no clear definition of UC or CD, it could be prone to such errors, especially for pediatric patients leading to skewed results.

The causal link between AD and IBD could be explained by several potential mechanisms. Several AD risk genes like IL1RL1, IL18R1, IL18RAP, ADAD1, KIAA1109, LRRC32, STAT3, RTEL1, ZGPAT, SLC9A4, IL13, C11orf30, TNFRSF6B, IL2/IL21 have also been linked with IBD suggesting a genetic relationship between the two [27]. Mendelian randomization studies have also found a strong causal association between AD and IBD [8, 28]. Second, both AD and IBD are diseases with altered barrier functions causing increased permeability, transport of pathogens, and hence inflammation [14]. Third, research shows that symptoms of AD in mice are relieved by provoking C‐C motif chemokine receptor 4 (CCR4) which is found to be increased in IBD as well [29, 30]. Evidence also shows that C‐C motif ligand 17 (CCL17), the chemokine ligand of CCR4 is required to induce colitis in mice [31]. CCL17 expression is significantly upregulated in AD [32]. Thus, these ligands may be an important link in the relationship between AD and IBD. Fourth, both AD and IBD have their pathogenesis linked with an inability to maintain a homeostatic relationship with the microbiota [33, 34]. Fifth, the Th2 immune pathway (namely Th1, Th17, and Th22 cells) plays a role in the development of AD [35]. Likewise, both Th1 and Th2 pathways have been implicated in IBD [36]. While these mechanisms are still under research, there is no single established causal link between the two diseases. There is a need for further research on pathophysiological mechanisms that can be targeted by therapeutic interventions and hence reduce the risk of IBD.

Some limitations of this review need to be noted. The evidence for this study comes mainly from registry data, which, while helpful in increasing the sample size, may suffer from problems such as errors or approximations in the processing of the data. Also, the method of diagnosis was not accurately recorded and the studies used mostly medical codes for identifying both AD and IBD. Diagnostic discrepancies are possible which could have affected results. Due to data limitations, we were unable to stratify the results by age, gender, and AD severity, which is a significant limitation as factors such as female sex and high disease activity are known to increase the risk of AD and other IMIDs. Lastly, several confounders were not measured in the included studies and hence residual confounding is a possibility.

Our review has important clinical implications. Current evidence shows that AD can lead to an increased risk of both CD and UC and physicians must be aware of the association between the two diseases. AD patients should be closely monitored for bowel symptoms and adequately counseled on the future risk of IBD. Also, there is a need for research on therapeutic options that can alter this risk and hence reduce the burden of IBD in AD patients.

## 5 Conclusions

Meta‐analysis from a large sample size of 61 million participants shows that AD is a risk factor for IBD. It significantly increases the risk of both CD and UC. Physicians should closely monitor AD patients for symptoms of IBD and provide appropriate and timely therapeutic interventions. Further research should be directed at establishing the underlying mechanisms linking IBD and AD.

## Ethics Statement

The authors have nothing to report.

## Consent

The authors have nothing to report.

## Conflicts of Interest

The authors declare no conflicts of interest herein.

## Supporting information


Figure S1.

Figure S2.

## Data Availability

The datasets used and/or analyzed during the current study are available from the corresponding author upon reasonable request.
