# Supplementary figures and images for: The Association Between Atopic Dermatitis and Inflammatory Bowel Disease Risk: A Meta‐Analysis of Longitudinal Studies
Source: JGH Open. 2024 Dec 12;8(12):e70077. doi: 10.1002/jgh3.70077 (PMC11638360; doi:10.1002/jgh3.70077)

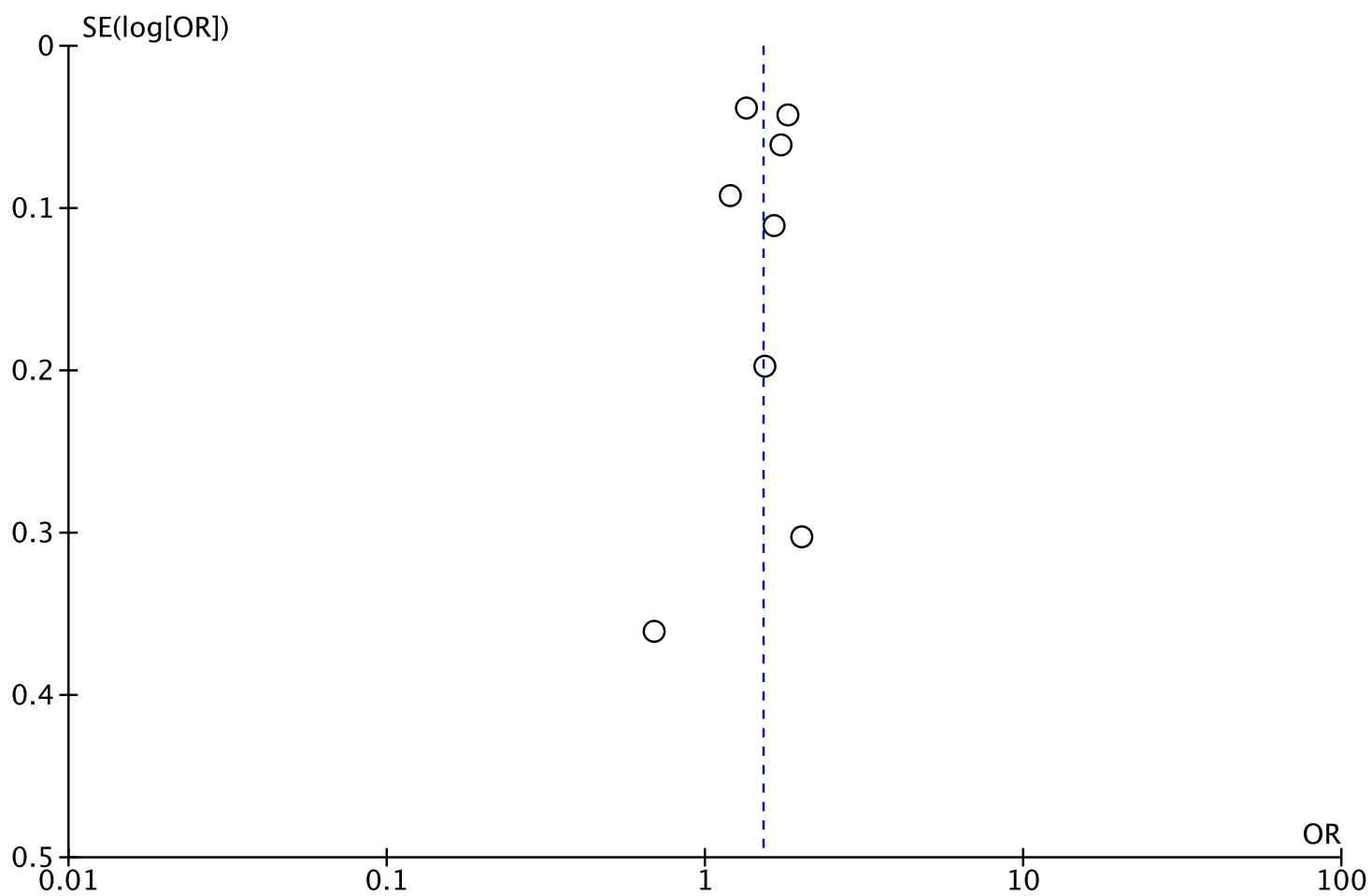

Supplement: Supplementary file 1 — Figure S1. [file JGH3-8-e70077-s001.pdf]

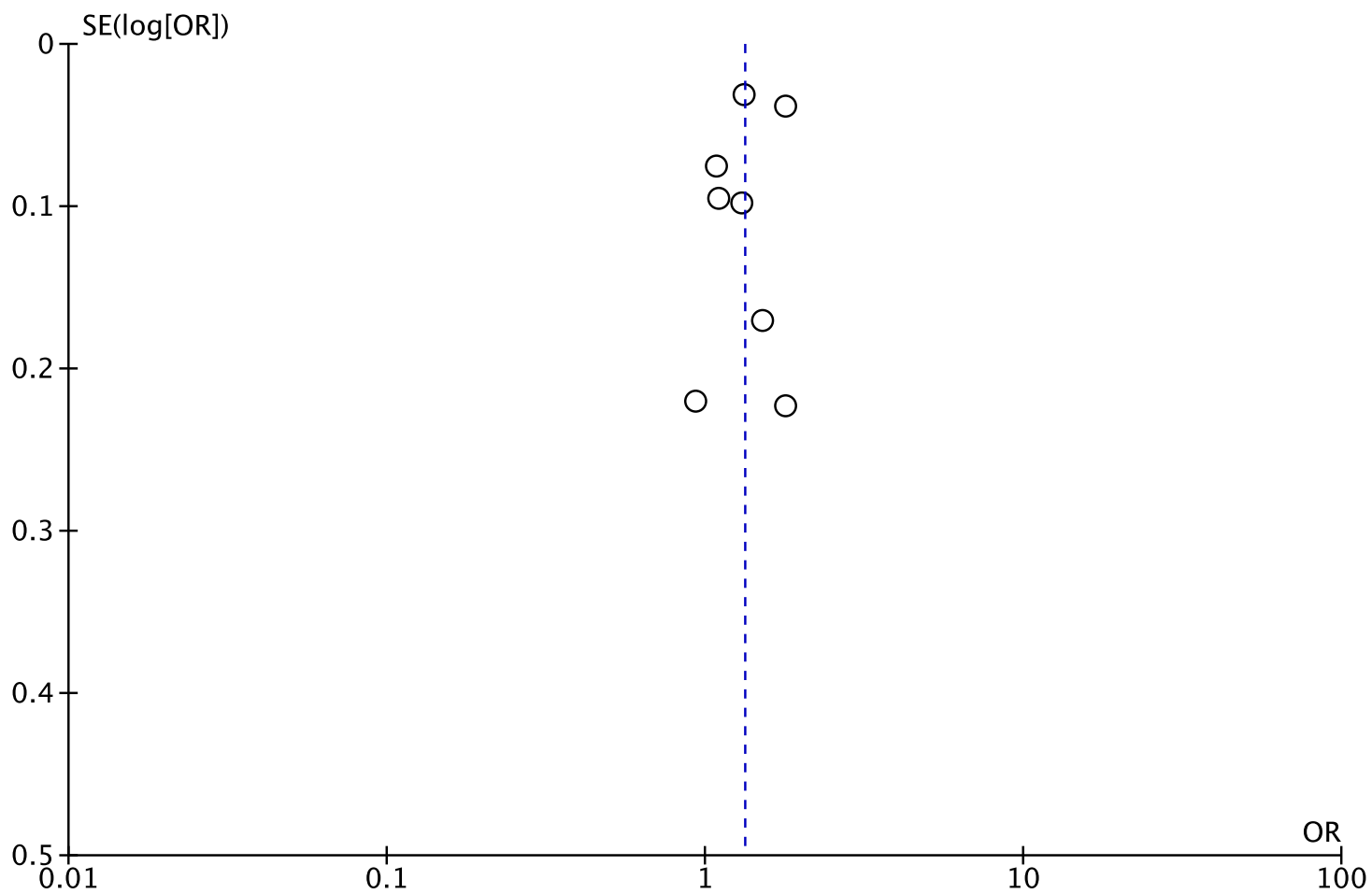

Supplement: Supplementary file 2 — Figure S2. [file JGH3-8-e70077-s002.pdf]
